# Validation of the Hungarian version of the Cognitive Failures Questionnaire (CFQ)

**DOI:** 10.1016/j.heliyon.2023.e12910

**Published:** 2023-01-10

**Authors:** Márta Volosin, Emese Hallgató, Eszter Csábi

**Affiliations:** a Institute of Psychology, University of Szeged, H-6722 Szeged, Egyetem utca 2, Hungary; b Institute of Cognitive Neuroscience and Psychology, Research Centre for Natural Sciences, H-1117 Budapest, Magyar tudósok körútja 2, Hungary

**Keywords:** Cognitive failures questionnaire (CFQ), Validation, Anxiety, Depression, Cognitive failures

## Abstract

Cognitive Failures Questionnaire (CFQ) is a widely utilized tool to measure the frequency of everyday cognitive lapses. Here we present a validation study of the Hungarian translation of CFQ. A subsample (n = 157) filled out the questionnaire twice within a 7–21 days interval to determine test-retest reliability. Exploratory structural equation modeling (ESEM) method was run on a larger sample (n = 382) for testing a different number of potential factors. Although the multiple-factor solutions showed good fit, the one-factor solution described the data more adequately. The composite reliability of the final model (CR = .822) as well as test-retest reliability (intraclass correlation coefficient = .900) and the internal consistency (Cronbach’s alpha = .920) of the CFQ were high. Higher CFQ scores (i.e., more cognitive slips) correlated positively with anxiety and depression while a negative relationship was present with well-being. Furthermore, women were characterized with higher CFQ scores compared to men. Our results are in line with previous studies, and the excellent psychometric properties make the Hungarian version of CFQ an appropriate measure of cognitive failures.

## 1 Introduction

Cognitive slips or failures can be defined as errors occurring during a task or activity that is usually executed by a person successfully [1]. Such failures are prevalent experiences in everyday life: for example, we might fail to remember someone’s name, turn in the wrong direction on a familiar road or fail to find an object which is in front of us. They might be manifested in perception, memory, or action, and their common feature is that “there is a departure from the normal smooth flow of function, and events do not proceed in accordance with intention” [2]; p. 1). That is, cognitive failures are linked to typical or automatically triggered, habitual activities rather than to actions requiring certain cognitive or motor abilities [3]. This is in line with the observation that the relationship between self-reported data and performance measured on objective tasks is rather inconsistent (for a review see: [4]), as objective tasks usually target cognitive functions requiring self-monitoring and control, and test stimuli are often characterized with poor ecological validity. Ref. [5] introduced three categories of action slips based on schema theories: errors might occur when forming intentions, in case of faulty, mainly data-driven activation of schemas, or due to faulty triggering like an intrusion of thought or spoonerism. On the other hand, cognitive failures can be explained in the context of automatic and controlled processes of attention originating from frontal lobe functions: attention lapses or inattention might lead to interference errors or reduced intentionality, while over-attention results in omissions, repetitions or reversals of an action [6]. Importantly, regardless of their origin, such everyday mistakes rarely occur in laboratory settings, therefore self-report measures might be crucial to quantify them [7].

To assess everyday cognitive slips, Ref. [2] developed the Cognitive Failures Questionnaire (CFQ) which consists of 25 items describing failure episodes that are common in everyday life, for example: “Do you forget where you put something like a newspaper or a book?” or “Do you leave important letters unanswered for days?” or “Do you fail to notice signposts on the road?”. Respondents have to rate the frequency of occurrence of each mistake in the last six months on a 5-point Likert scale where 0 corresponds to “never” and 4 corresponds to “very often”; the theoretical minimum is 0 and the theoretical maximum is 100. Samples of healthy younger or middle-aged adults indicate average scores ranged typically between 30 [8,9] and 45 points [10]. CFQ is a widely utilized and accepted measure which is reflected by the fact that it has been translated to different languages, for example, Hebrew [11], German [12], Dutch [13], Italian [14], Brazilian [15] or Turkish [16].

The different studies used various methods and populations to assess the construct validity of CFQ, especially when defining the number of factors of the questionnaire. In the original study Ref. [2], suggested that cognitive failures represent a single factor, and this finding has been reinforced later [10,13,17,18]). In contrast, other studies demonstrated 2–7 components or factors underlying CFQ scores. Refs. [8,9] suggested a 2-component solution on a sample of N = 2379 navy personnel: one component for general cognitive failures and one minor component for name processing. Utilizing the German version of CFQ which included 7 additional items on a sample consisting of students, library and dry cleaner staff (N = 213), distractibility and sensory-motor coordination emerged as the two dominant components [12]. The Dutch version of CFQ [13] demonstrated a 3-component solution on a representative sample of N = 1303 adults from 24 to 83 years: forgetfulness, distractibility, and false triggering [19]. A 4-component solution was proposed by Ref. [20] based on data of N = 335 respondents from university students and navy personnel: memory, distractibility, blunders, and names. Three further studies suggested a 5-component structure of CFQ: based on data of N = 387 university students, distractibility, misdirected actions, spatial/kinetic memory, memory for names, and interpersonal intelligence were proposed [7]; and based on a navy sample (N = 535) Ref. [21] suggested the presence of a factor for the general failure of attentiveness and memory, for social interaction, for motor/spatial, emotional regulation and for control over pursuit goals. The Turkish version of CFQ [16] applying a 4-point Likert scale also revealed 5 components: general cognitive failures, inattention, concentration, names, and social failures. Finally, the Italian validation on a sample of N = 830 undergraduate respondents suggested the presence of 7 components: action monitoring and control, memory for names, decision making, mind-wandering, over-reliance of memory, blunders and absent-mindedness [14].

In most of the above-mentioned studies, components were extracted on the basis of eigenvalues being higher than 1.00, and researchers utilized principal component analysis (PCA) with either Varimax [[7], [8], [9],[12], [13], [16],^20^] or Oblique rotation [10,14,19,21]. The following pattern of eigenvalues was typical: the first extracted factor or component had a relatively high eigenvalue while the eigenvalues of further components were around 1.00 [[7], [8], [9],^21^]. When comparing compared Kaiser’s extraction method with PA directly [12], found that while eigenvalues were above 1.00 in 11 cases, PA only revealed two components. This further indicates that defining components based on eigenvalues >1.00 might lead to over-extraction [22].

Furthermore, and more importantly, the majority of these validation studies were exploratory without any further confirmation of the factor structure which can also contribute to the different number of factors extracted on different samples. Only a few studies applied confirmatory factor analysis (CFA) method [3,18,19,21]). Ref. [19] tested the one-factor solution of [2]; the two-factor solution of Ref. [8,10]; the four-factor solution of Ref. [20]; and the five-factor solution of Ref. [7]. Additionally, they applied EFA and CFA on independent samples which indicated a three-factor solution. While the one-, two-, four- and five-factor models showed nearly identical acceptable fit (comparative fit index (CFI) between .95 and .96; root mean square of approximation (RMSEA) between .071 and .066), the three-factor model overperformed all of them (CFI = .97, RMSEA = .056). In contrast, when Ref. [3] compared the single-factor, two-factor [8,9], four-factor [11,20] and five-factor [7] models, neither model showed good fit of the data (CFI between .556 and .720; RMSEA between .070 and .116); finally, the authors suggested a 12-item version featuring three factors. Very similarly, comparing the one-factor solution of Ref. [2]; the four-factor solution of Ref. [20] and a further five-factor solution resulted from EFA, low fit results were present (CFI between .85 and .88; RMSEA = .07) [21]. Using Rasch model, Ref. [18] found one large general and five narrow factors, and although the six-factor solution showed better fit, the author suggested the dominance of a general factor of cognitive failures. Similarly Ref. [17] tested the one-factor solution of Ref. [2]; the three-factor solution of Ref. [19] and the four-factor solution of Ref. [20]; and they found that although Ref. [19] factor solution showed the best fit, it suffered from several discrepancies such as extremely high correlations between factors or negative factor loadings. However, when the covariance between items was corrected, the one-factor solution had as high fit indices as multi-dimensional solutions along with a more interpretable structure, emphasizing the superiority of the one-dimensional solution [17].

A further critical step in validating questionnaires is to assess the internal consistency and reliability. The internal consistency of CFQ indicated by Cronbach’s alpha was found to be high: between .75 [13] and .94 [17], suggesting that items are closely related to each other. The test-retest reliability was measured less often but it also tended to be high: in case of shorter periods (in 1–6 weeks) between the two occasions of data collection, correlation coefficients ranged between .81 [15] and .83 [13]. When the time interval between the two data collection was longer, such as 2 or 8 years, the correlation coefficients were still satisfactory: *r* = .71 and *r* = .73, respectively [21,23].

Besides investigating the factor structure and internal consistency of the questionnaire, relationship with further related psychological constructs is also important to be mapped. Several studies found a positive correlation with trait [[12], [13], [16], [17],^18^,^24^,^25^] or test anxiety [26], neuroticism [2,3,10], depressive symptoms [[13], [16], [17],^27^], and a negative association with coping [12]. Negative affect correlated positively with memory and attention failures reported on CFQ and positive affect correlated negatively with CFQ items on distractibility [28]. Stress [2,21,24] and boredom proneness [20,29] were also found to be predictive of CFQ scores. Besides, tense mood was found to enhance the effect of fatigue on reporting cognitive failures [30], and enhanced daytime sleepiness [29] and insomnia [31] were related to higher CFQ scores.

Demographic factors such as gender or age also influence the proneness to report cognitive failures both as stable or variable dimensions [4]. There is evidence that women tend to report more [21,32,33] or a similar number of cognitive failures [28] than men. Results on aging, however, are not equivocal, despite that the vast majority of studies investigated psychometric correlates of CFQ on samples of young adults. As aging is associated with deteriorated frontal lobes functions, resulting in a decline in inhibitory mechanisms, attention [34], and memory (for review see: [35,36], it is reasonable to assume that the frequency of self-reported cognitive failures increases with age. In contrast, studies failed to demonstrate differences in CFQ scores between younger and older adults [37,38], or within older age groups [23,39,40]. More surprisingly, older age was associated with fewer slips being reported in comparison to younger age groups [16,33,41,42]. This paradox could be explained by the fact that older adults might forget some of their cognitive failures [41] and their ability to monitor and report absent-mindedness might decrease [33,41]. This notion can be easily validated by utilizing reports from informants: while younger adults tended to underestimate their cognitive functioning, older adults systematically overestimated them relative to informant reports [42] which is in line with the results of Ref. [19] who demonstrated that people reported being more forgetful but less distractable with age starting around their sixties. In fact, older adults who are aware of their cognitive abilities often use external compensational strategies [4,42] such as notes or reminders [43] which also contribute to the underreporting of cognitive failures. Finally, retirement-related lifestyle changes lower the attentional demand in general, resulting in a lower risk of cognitive failures [33,41].

To summarize, although results on construct validity and reliability of CFQ are consistent in general, there is less agreement about its factor structure which can be explained by the dominance of the exploratory nature of methods and the use of different criteria in assessing its factor structure. The goal of the present study was to validate CFQ on a Hungarian sample by assessing its internal structure and construct validity. First, we mapped the underlying factor structure by Exploratory Structural Equation Modeling (ESEM) [44] on a larger sample of healthy adults (Sample I). Internal consistency and factor invariance of gender were also tested. Second, on a smaller subsample (Sample II) we calculated test-retest reliability. Our third goal was to investigate relationship with anxiety, depressive symptoms and well-being, and to explore associations with age and gender. We hypothesized higher CFQ scores for women compared to men, a positive relationship between CFQ and anxiety and depression, and a negative relationship with age and well-being.

## 2 Material and methods

### 2.1 Participants

Hungarian participants over 18 years were recruited by convenience and snowball sampling. They were required to fill out an online questionnaire once or twice (see below). As older populations are far more difficult to reach online, some of the respondents older than 60 years (n = 20) filled the questionnaire on paper, and their responses were coded identically to those participating in the online study.

A total of 576 respondents completed the questionnaires at least one time. As our goal was to validate CFQ on a healthy sample, and given that several illnesses and medications might affect cognitive performance, respondents with the following diagnoses were excluded: endocrine disorders (e.g. hypo- or hyperthyroidism) and certain types of autoimmune diseases (e.g. intestinal diseases, allergies with antihistamine medication), PCOS, diabetes, insulin resistance; psychiatric problems (e.g. anxiety, depression or bipolar depression, panic disorder, PTSD); neurological problems (e.g. sclerosis multiplex, severe hearing or vision impairment, memory impairment); cardiovascular diseases (high or low blood pressure, heart disease); cancer; or acute illness (e.g. Covid-19). We also excluded participants who reported that they had a diagnosis or were under medication but did not provide further details on this. On the other hand, respondents with health conditions or medications without known interfering effect with cognition were not excluded, e.g., those with psoriasis, asthma or allergies without medication, eczema, celiac or dairy intolerance, spinal problems (e.g., scoliosis), reflux, anaemia, myopia, cartilage abrasion, prostate enlargement, endometriosis or obesity. Participants who were taking nutritional supplements, contraceptives or vitamins were also included in the final sample.

As a result, the final sample (Sample I) consisted of 382 participants (119 men and 262 women, one not responded). Mean age was 33.40 years (Med = 30, SD = 12.53, from 18 to 70 years), and the average time spent in education was 16.47 years (Med = 16, SD = 3.23, from 5 to 40 years). Descriptive statistics of age and education separately for men and women are presented in Table 1.

**Table 1:** Descriptive statistics of the samples regarding age and education in years in the sample for the single and repeated data collections (Sample I and Sample II).

			Mean	Median	SD	Minimum	Maximum
Sample I	Age	Men	33.62	31	12.54	18	86
		Women	33.20	30	14.48	18	80
	Education	Men	16.14	16	2.80	8	24
		Women	16.61	16	3.41	5	40
Sample II	Age	Men	35.82	32	15.57	18	68
		Women	32.87	30	12.62	18	70
	Education	Men	15.27	14	3.35	8	24
		Women	16.29	16	3.90	8	40

In order to and to measure test-retest reliability and temporal consistency of CFQ, a smaller subsample of participants was drawn from the above mentioned 576 respondents. They completed the test battery twice, the second occasion being within 1–3 weeks after the first one. To identify matching responses, each respondent was given a unique identifier which they had to use for both data collections. A total of 474 completions were received between March and April of 2021. In the case of 177 identifiers, two completions were present as instructed and as expected. In the case of 68 identifiers, the corresponding participants completed the questionnaires only once, and there were 15 participants who completed the questionnaires three times, typically two completions occurring on the same day and one completion 1–3 weeks after. In these cases, the first completion of the same-day completions was included in the data analysis. In addition, we found 3 identifiers with 4 completions. By looking at the demographic characteristics, we revealed that in two cases two different participants used the same identifiers, and they completed the questionnaires twice (as expected). Their responses could be clearly distinguished from each other, and were included in data analysis. Regarding the remaining one identifier with 4 completions, two completions were found to originate from a single participant, and these were included in the data analysis, while the remaining two completions did not match with any other completions, and were thus excluded. This resulted in 195 valid pairs of responses.

In the next step, we examined whether the demographic data were meaningful and identical (or nearly identical) across the two completions. All respondents who did not give an exact numerical answer to the question on education in years in their lifetime (for example, who answered “a lot” or “university degree”), and those whose age or education in years differed by more than one between the two completions, as well as those who gave different residence or gender across the two completions, were excluded from further analysis. Given that differences between individuals were not subject of interest in this step of analysis, we did not exclude any participants based on their health status.

As a result, the test-retest reliability analysis was conducted on a total of 157 participants (51 men, 106 women – Sample II) who filled out all questionnaires twice. Mean age was 33.38 years (Med = 30, SD = 13.70 from 18 to 70 years), and the average time spent in education was 15.96 years (Med = 16, SD = 3.75, from 8 to 40 years). The median number of days between the two completions was 11 days (M = 11.32, SD = 3.26). Descriptive statistics of age and education separately for men and women are presented in Table 1. In addition to the test-retest reliability, we conducted an item-wise analysis as well in order to examine whether there are any items which seem to be unstable over time. The steps and results of the item analysis are presented in Supplementary A.

The study was conducted in accordance with the Declaration of Helsinki and the protocol was approved by the United Ethical Review Committee for Research in Psychology, Hungary (EPKEB; Reference number: 2021–07).

### 2.2 Instruments

Before filling out the questionnaires, participants gave an informed consent. The questionnaires started with demographic questions (e.g., gender, age, level of education, type of settlement, health condition), followed by the psychological questionnaires detailed below. The completion of the questionnaires took approximately 20–25 min. We would like to note that this study was conducted as a part of a project during which not only the Hungarian version of the CFQ but also the Multifactorial Memory Questionnaire (MMQ – [45] was aimed to be validated, so MMQ was included into questionnaire battery as well. However, as the focus of the present paper is to report the validation of CFQ, results on MMQ are reported elsewhere (Csábi et al., under review).

#### 2.2.1 Cognitive Failures Questionnaire (CFQ)

CFQ was utilized to measure the prevalence of the cognitive failures in respondents on a 5-points Likert scale from 0 to 4 in which higher values indicate a higher frequency of cognitive failures in the last six months. The Hungarian translation of the CFQ [2] was performed independently by two of the authors (ECs and MV). These two translations were compared and adjusted by all of three authors resulting in a finalized version which was translated back to English by a Hungarian-English bi-lingual person who was not familiar with the original version of CFQ. Finally, the English version resulting from this translation and the original English version were compared, and their similarity was considered to be satisfactory by two individuals with proficient English skills. As there are no substantial cultural differences between Hungary and the United Kingdom which would have affected any of the items of the questionnaire, no culture-specific adaptation was needed. The Hungarian translation along with the original CFQ are presented in Supplementary B.

#### 2.2.2 World Health Organization Five Well-being Index (WHO-5)

The WHO-5 [46] is a 5-item questionnaire of current mental well-being. In the Hungarian version [47] respondents have to rate the perceived frequency of each item in the past two weeks on a 4-points Likert scale (0: at no time; 3: all of the times). Higher scores represent higher well-being.

#### 2.2.3 Patient Health Questionnaire (PHQ-9)

The aim of the PHQ-9 [48] is to assess the frequency of 9 depressive symptoms over the past two weeks. Respondents have to indicate on a 4-points Likert scale the frequency of the occurrence of each symptom (0: not at all – 3: nearly every day). Besides, in case of the occurrence of at least one symptom, they also have to rate the level at which it caused difficulties in work or household or in their social relations. Higher scores indicate a higher level of depressive symptoms.

#### 2.2.4 Beck Anxiety Inventory (BAI)

In order to assess anxiety, we utilized the Hungarian version of the BAI [49,50] which consists of 21 items. Respondents have to rate on a 4-points Likert scale the degree at which different anxiety-related symptoms bothered them during the last week (0: not at all – 3: severely). Higher scores indicate higher anxiety.

### 2.3 Statistical analyses

First, on Sample I, the factor structure of CFQ and factorial invariance of gender were assessed by Exploratory Structural Equation Modeling (ESEM) on the remaining items. ESEM is a strategy developed by Ref. [44] which compromises between the flexibility of exploratory factory analysis (EFA) and the rigor of confirmatory factor analysis (CFA) within the same solution [51] by transforming factor loading matrix resulted from EFA into structural equations which can be further tested on the same sample [52]. As there is no equivocal agreement in the literature on the numbers of factors or latent variables measured by CFQ, we aimed to find the best fitting factor structure by testing ESEM models of 1, 2, 3, 4, 5, 6 and 7 factors. We applied ESEM the procedure as suggested by Ref. [52] and models were estimated using maximum likelihood estimator and geominQ rotation. Model fit was evaluated by the following indices: Chi-squared (*Χ*^*2*^), normed Chi-squared (*Χ*^*2*^*/df*), comparative fit index (CFI), Tucker Lewis fit index (TLI), root mean square of approximation (RMSEA), and standardized root mean square residual (SRMR). Values for *Χ*^*2*^*/df* < 2 [53], CFI and TLI > .95, RMSEA < .06 and SRMR < .08 were indicating good fit [54,55]. Higher CFI and TLI values along with lower RMSEA, SRMR, *Χ*^*2*^ and *Χ*^*2*^*/df* values indicate improvement in model fit. It is important to highlight that although these cutoff values are widely accepted, it was pointed out that such strict a priori criteria should be interpreted cautiously [54,55], and they easily lead to enhanced Type I errors, that is, to incorrect rejection of an otherwise acceptable model [51].

After the most appropriate factor structure was defined, factorial invariance [52,56] for gender was assessed and composite reliability (CR [57,58]); was calculated for the best fitting model, as well as Cronbach’s alpha and test-retest reliability were calculated for the scale. For the latter, we used intraclass correlation (ICC) coefficient [59]. Finally, construct validity was investigated by Spearman’s correlations with depressive and anxiety symptoms and well-being. Spearman’s correlation was also used to reveal relationship with age, and gender differences on CFQ scores were calculated on data from single data collection. Descriptive statistics, correlations and Cronbach’s alpha were calculated in jamovi 2.2.5 [60]. ESEM and ICC calculations were performed using R statistical software (version 4.0.5 [61]). For aggregating data, data. table (version 1.14.0 [62]) package; for factor analysis and computing ICC, psych (version 2.1.3 [63]) package, and for computing ESEM models, factorial invariance and CR, lavaan (version 0.6.12; [64]) semTools (version 0.5.6 [65]) and ccpsyc (version 0.2.6 [66]) packages were used.

## 3 Results

### 3.1 Factor structure

We conducted 1, 2, 3, 4, 5, 6 and 7-factorial ESEM models in order to assess the best fitting structure. Factor analysis results are summarized in Table 2.

**Table 2:** Fit indices for each model resulted with ESEM.

Model	*Χ*^*2*^*(df)*	*Χ*^*2*^*/df*	CFI	TLI	RMSEA (90% CI)	SRMR	Cumulative variance (%)
Single-factor	915.89 (299)	3.06	.822	0.821	.074 (.068–.079)	.089	33
Two-factor	745.39 (300)	2.48	.870	0.869	.063 (.057–.069)	.074	36
Three-factor	605.50 (300)	2.02	.910	0.908	.053 (.047–.059)	.053	39
Four-factor	500.40 (290)	1.73	.939	0.937	.044 (.037–.050)	.044	42
Five-factor	410.87 (285)	1.44	.964	0.962	.034 (.026–.041)	.059	46
Six-factor	329.58 (279)	1.18	.985	0.984	.022 (.009–.031)	.055	48
Seven-factor	282.72 (272)	1.04	.997	0.997	.010 (.000–.023)	.051	50
Single-factor corrected	482.59 (257)	1.88	.929	0.924	.048 (.041–.055)	.074	33

Note: *Χ*^*2*^*/df* = normed Chi-squared, CFI = comparative fit index, TLI = Tucker Lewis fit index, RMSEA = root mean square of approximation, SRMR = and standardized root mean square residual.

As presented in Table 2, increasing the number of factors improved values of fit indicators, as well as the amount of explained variance, and solutions including five or more factors showed acceptable or good fit (CFI and TLI > .95, RMSEA and SRMR < .08). The five-factor solution is presented in Table 3. Based on the items assigned to each factor, Factor 3, 4, 1, 2 and 5 could be named “Distractibility”, “Memory and blunders”, “Interpersonal intelligence”, “Names” and “Sensory-motor coordination”. Between-factor correlations are presented in Table 4.

**Table 3:** The factor loadings of the five-factor solution of CFQ resulted from EFA. Items corresponding to certain factors are highlighted in bold. In the lower part of the Table, SS loadings, percentage of proportional and cumulative variances are presented.

Item number	Factor 3	Factor 4	Factor 1	Factor 2	Factor 5
1. Do you read something and find you haven’t been thinking about it and must read it again?	**0.989**	−0.348			
19. Do you daydream when you ought to be listening to something?	**0.884**				−0.264
21. Do you start doing one thing at home and get distracted into doing something else (unintentionally)?	**0.729**				−0.276
2. Do you find you forget why you went from one part of the house to the other?	**0.569**	0.157			0.204
15. Do you have trouble making up your mind?	**0.568**				
22. Do you find you can’t quite remember something although it’s “on the tip of your tongue”?	**0.427**			0.255	
11. Do you leave important letters unanswered for days?	**0.422**	0.260			
14. Do you find yourself suddenly wondering whether you’ve used a word correctly?	**0.404**		0.174		0.132
25. Do you find you can’t think of anything to say?	**0.386**	0.204			
3. Do you fail to notice signposts on the road?	**0.325**		0.134		0.235
10. Do you lose your temper and regret it?	**0.322**		0.267	0.116	
6. Do you find you forget whether you’ve turned off a light or a fire or locked the door?	**0.284**	0.139	0.181		
17. Do you forget where you put something like a newspaper or a book?		**0.738**			
16. Do you find you forget appointments?		**0.711**			
24. Do you drop things?	0.203	**0.512**			
23. Do you find you forget what you came to the shops to buy?	0.157	**0.492**			
13. Do you fail to see what you want in a supermarket (although it’s there)?	0.297	**0.413**			
18. Do you find you accidently throw away the thing you want and keep what you meant to throw away – as in the example of throwing away the matchbox and putting the used match in your pocket?		**0.405**	0.106		0.110
9. Do you fail to hear people speaking to you when you are doing something else?		**0.315**	0.213		
12. Do you find you forget which way to turn on a road you know well but rarely use?	0.171	**0.301**			0.161
8. Do you say something and realize afterwards that it might be taken as insulting?			**0.993**		
20. Do you find you forget people’s names?				**0.962**	
7. Do you fail to listen to people’s names when you are meeting them?	0.175	−0.147	0.214	**0.457**	
4. Do you find you confuse right and left when giving directions?	0.148				**0.494**
5. Do you bump into people?		0.290	0.179		**0.380**
SS loadings	4.720	3.010	1.550	1.460	0.810
Proportion variance	0.190	0.120	0.060	0.060	0.030
Cumulative variance	0.190	0.310	0.370	0.430	0.460

**Table 4:** Between-factor correlations for the five-factor solution.

	Factor 3	Factor 4	Factor 1	Factor 2	Factor 5
Factor 3	1.00				
Factor 4	.73	1.00			
Factor 1	.45	.38	1.00		
Factor 2	.37	.36	.28	1.00	
Factor 5	.35	.31	.16	.15	1.00

However, there are at least two concerning facts about the five-factor solution. First, Factor 1 consists of one item only while Factor 2 and Factor 5 consists of two items each. Second, item 6 loads on multiple factors with low loadings (below 0.3) which is problematic when interpreting the factor. Third, the range of correlation coefficients between factors is broad: the lowest correlation coefficient is .15 while the highest is .73.

Therefore, based on the solution and suggestions of Ref. [17]; in the next step we explored the scree plot of eigenvalues of CFQ: the first factor demonstrated a high eigenvalue (above 8) while the other eigenvalues were below 1, indicating that a one-dimensional solution should be considered. Following this, we inspected the modification indices of the one-factor solution, and corrected the model with items where modification indices were higher than 10. This way the covariance of item pairs with similar content is included into the model which improves the fit [17,67]. After this correction, fit of the one-factor model improved substantially, however, not all of the fit indicator values exceeded the a priori defined thresholds of good fit (Table 2). Despite of the moderate fit indices, based on the inspection of scree plot and the concerning factor structure of the five-factor solution (low number of items on factors and low factor loadings), we choose the corrected one-factor solution for defining the internal structure of CFQ, and to assess factorial invariance of gender.

Configural invariance analysis of gender showed slightly lower fit than the model without including gender: CFI = .892; RMSEA = .060 (for a comparison, see Table 2). In case of metric invariance, no difference was present from configural invariance (CFI = .892; RMSEA = .060), suggesting gender invariance when factor loadings were forced to be equal. However, scalar invariance where in addition to factor loadings, factor item intercepts are forced to be equal, significantly differed from metric invariance (CFI = .880; RMSEA = .062; ΔΧ^2^ = 23; ΔCFI = .013; ΔRMSEA = .002). Importantly, as ΔCFI larger than -.01 suggests that null hypothesis on invariance should be rejected [56], the present results indicate that invariance for gender was not proven.

Finally, composite reliability (CR) of the final CFQ model as well as Cronbach’s alpha and test-retest reliability of the scale were calculated. CR of the model was .822, Cronbach’s alpha was .920 and ICC was .900 which suggest excellent internal consistency and test-retest reliability.

### 3.2 Psychometric correlates

Before assessing correlates of CFQ with anxiety, depression and well-being, measurement validity and internal consistency of BAI, PHQ-9 and WHO-5 were calculated on our data. Confirmatory factor analysis indicated excellent or acceptable fit indices for PHQ-9 (*Χ*^*2*^ (27) = 57.911, *p* < .001; CFI = .969, TLI = 0.959; SRMR = .036; RMSEA = .055) and for WHO-5 (*Χ*^*2*^ (5) = 24.629, *p* < .001; CFI = .967, TLI = 0.933; SRMR = .035; RMSEA = .102). However, the fit of BAI was poor to the present data set: *Χ*^*2*^ (189) = 1068.251, *p* < .001; CFI = .733, TLI = 0.703; SRMR = .073; RMSEA = .110. When correcting the BAI model with items where modification indices were higher than 10, model fit indices became satisfactory: *Χ*^*2*^ (189) = 348.908, *p* < .001; CFI = .944, TLI = 0.929; SRMR = .049; RMSEA = .054, therefore we decided to keep BAI for further correlational analysis. Both composite reliability of the models (CR for PHQ-9 = .845, for WHO-5 = .807, and for BAI = .872) as well as the Cronbach’s alpha of the scales (PHQ-9 = .839, for WHO-5 = .805 and for BAI = .907) suggested good or excellent internal consistency for all three measurements. Summarized scores of CFQ correlated positively with anxiety (*r*_*s*_ (380) = .506, *p* < .001) and depression symptoms (*r*_*s*_ (380) = .535, *p* < .001), suggesting that individuals who described themselves as more depressive or anxious, reported a higher prevalence of cognitive failures. In contrast, well-being (*r*_*s*_ (372) = -.357, *p* < .001) was negatively associated with the amount of cognitive failures being reported.

Age (*r*_*s*_ (380) = -.197, *p* < .001) was negatively associated with the amount of cognitive failures being reported. However, as aging is often associated with enhanced level of depression and anxiety manifesting in pseudodementia [68], we conducted partial correlations to reveal a more straightforward relationship between these variables and CFQ scores. When the effect of age was controlled, the significant relationship between CFQ and anxiety (*r*_*s*_ (380) = .482, *p* < .001), depressive symptoms (*r*_*s*_ (380) = .508, *p* < .001) and well-being (*r*_*s*_ (372) = -.349, *p* < .001) was still observable. More importantly, when anxiety, depression and well-being were controlled, the relationship between age and CFQ scores disappeared (*r*_*s*_ (372) = -.027, *p* = .600), suggesting that CFQ might rather reflect a general worry about cognitive abilities.

Because of the skewed distribution of CFQ scores (males: Shapiro-Wilk’s *W* = 0.950, *p* < .001; females: *W* = 0.957, *p* < .001), gender differences were investigated using the Mann-Whitney test. We found that women tended to report significantly higher number of cognitive failures than men (*U* = 12154.5, *p* < .001; Med_females_ = 24 vs Med_males_ = 29).

## 4 Discussion

The goal of the present study was to validate the Hungarian version of the CFQ by assessing its internal structure validity and construct validity. Although CFQ showed acceptable fit in case of at least five factors, irrespectively of the good fit values of the multi-dimensional models, it was more appropriate to describe CFQ as a single-factor scale, therefore participants were characterized with a single value resulting from summing up their responses. CFQ had an excellent internal consistency as well as good test-retest reliability. Furthermore, higher CFQ scores were associated with higher anxiety, more depressive symptoms and lower well-being. Besides, CFQ scores were negatively associated with age and were lower in men compared to women.

The five-dimensional factor structure of CFQ is comparable with studies of Refs. [7,16,21] as they also suggested five-factor solutions. However, the content and naming of factors are not identical, and when comparing the items of different factors from other studies suggesting multiple factors, there are both overlaps and inconsistencies among the interpretation of the factors. In addition to the obvious differences in factor extraction methods, sample size and sample characteristics, finding an equivocal factor structure is also difficult because certain items loaded to multiple factors with relatively high loadings (in the present study, see Table 3., but also see Refs. [[7], [8], [9], [10],^16^,^19^,^18^,^15^]. Along with the correlating factors, this suggests that although cognitive failures can be regarded as a complex construct, its sub-dimensions cannot be clearly separated. This notion is supported by a very recent critical study of Ref. [17] who suggested that the previously reported multidimensional factor structures are misleading and CFQ should be used without any subscales. Nevertheless, the original version of the questionnaire was designed to measure one general factor of cognitive failures [2], and one-component solutions were demonstrated on Dutch [13] and US samples [18]. Furthermore, Ref. [10] pointed out that CFQ has insufficient items to measure more than two strongly defined components.

Despite of the various number of factors demonstrated in the above-mentioned studies, CR, Cronbach’s alpha values and test-retest reliability were calculated for the whole scale and not for the individual components. The present study demonstrated high CR for the corrected single-factor CFQ model, and also showed Cronbach’s alpha of .920 and test-retest reliability measured by ICC of .900. This fits well to previous results demonstrating Cronbach’s alphas between .75 and .93, and test-retest reliability (Pearson’s rs) between .71 and .83. This suggests that irrespective of the underlying component structure, the internal consistency and test-retest reliability of CFQ can be considered as high. Nevertheless, it is important to note that extremely high Cronbach’s alpha values (above .900) reflect that some items are redundant and they are testing the same question [69,70].

Our results regarding the relationship with depressive and anxiety symptoms and well-being supported our hypotheses. Respondents with higher anxiety and depression as well as those who reported lower well-being reported more cognitive failures, in line with previous studies [[12], [13], [16],^21^,^18^]. Although the direction of the causality cannot be clearly demonstrated, this might suggest that proneness to admit cognitive failures reflects general complains and worries about one’s cognitive abilities [3]. This is in accordance with the notion that negative emotionality is associated with higher proneness to complain about one’s health and mental abilities and with enhanced error-monitoring even during simple tasks [71]. On the other hand, experiencing cognitive failures and poor executive control were also found to be linked to everyday strain and to enhanced vulnerable to stress, leading to lower well-being and enhanced anxiety [10,21.

The relationship between reported cognitive failures and aging is not trivial. Despite the age-related decline in memory and executive functioning in general [34], aging was associated with lower CFQ scores. This fits to results suggesting that the poorer memory in older age might lead to failures being forgotten, and that a cognitively less demanding lifestyle after retirement lowers the amount of information that has to be remembered or attended to [33,41]. It is important to highlight that the subjective reports do not necessarily reflect actual executive skills which are measured in laboratory settings (e.g., Refs. [1,72,73]), and that dissociation between objective and subjective measurements or informant reports can be even higher when individuals start to be more forgetful and distractable [19,42]. Although we excluded all participants who reported diagnoses which could affect their cognitive abilities, given that these data were self-reported as well, there still might have been remained undetected conditions. Especially in the older respondents, data on their cognitive status indicated by dementia screening test such as Mini Mental State Examination (MMSE – [74]) would be informative. Therefore, and because subjective memory complaints are related to the pre-clinical stage of dementia [75], using CFQ in geriatric conditions should be considered.

More importantly, when controlling for age, the relationship between CFQ and anxiety, depression and well-being remained significant but when anxiety, depression and well-being were controlled, the age and CFQ were no longer associated. This resonates to the results of Ref. [76] who also demonstrated that controlling demographic variables did not affect the strong correlation between CFQ and depression, and indicates that the CFQ score reflects worries rather than objective problems in cognitive functioning, irrespective of age [76].

Finally, we demonstrated that women reported significantly more cognitive failures than men which is in line with previous studies [21,32,33]. This difference might originate from biological differences as well as from personality traits which are more prevalent in women [4]. For example, neuroticism and negative affect were found to be higher in women [3,28] which are associated with enhanced self-monitoring, leading to enhanced perception of cognitive failures and awareness of errors [4,77]. It is important to point out that not only biological or personality traits but societal role differences between men and women might add up to the higher CFQ scores of women. Women raising two or more children reported more cognitive lapses than those caring for a lower number of children, especially when emotional support was low [78]. Furthermore, women also tend to bear more invisible labour such as managing the household or caring for family members which was more pronounced during Covid-19 [79] when the data collection for the present study occurred. This implies that further studies should investigate the background of gender differences in cognitive failures at multiple levels.

The present study has both advantages and limitations. The first advantage is that we utilized ESEM which allows to combine exploratory and confirmatory methods on the same data set, providing more accurate results on the factor structure in comparison to a single exploratory analysis which was used in majority of validation studies. Second, in addition to the test-retest of the whole scale, we also inspected temporal stability of single items and flagged items which appeared to be less consistent across time (Supplementary A). On the other hand, it is a shortcoming that although our sample size was satisfactory, it cannot be regarded as representative, and neither the gender ratio or age were counterbalanced. Additionally, because older population is more difficult to reach online, a small sample of older adults filled questionnaire offline instead of an online form which could have led to inconsistencies between online and offline responses.

These limitations might negatively impact external validity, that is, the generalizability of the results to the representative healthy as well as to pathological populations. There is evidence in the literature that CFQ scores in certain clinical and sub-clinical groups might differ. Clinical groups with depression [13,16,80], anxiety [13,16] and traumatic brain injury [81] reported higher CFQ scores than the healthy controls while OCD patients reported lower scores [13]. Besides, positive correlation was demonstrated with ADHD-related symptoms [15], dissociative experiences, and schizotypy [82,83]. A possible explanation for the latter is that lapses in cognitive control trigger mild dissociation that is similar to those occurring during failures in routine tasks [4]. It was also demonstrated that sub-clinical checking compulsions were associated with impairment in prospective memory leading to enhanced experiences of cognitive failures, further intensifying intrusive thoughts on checking compulsions [84].

In addition, the utilization of objective measurements could further improve the predictive or criterion validity of CFQ, that is, the extent the test predicts outcomes of another related behavior [85]. Higher CFQ scores were predictive on traffic behavior by being associated with enhanced occurrence of accidents [8,9] and errors produced during simulated driving [14]. The frequency of everyday failures such as returning a book late to the library or losing objects showed a weak but significant relationship with CFQ scores as well [12]. Furthermore, CFQ scores were predictive of the treatment outcome of spider phobia [13]. Regarding intelligence or laboratory-related tasks, there are mixed results: there is data suggesting that intellect and abstract reasoning skills were associated with CFQ [18] but other studies found no relationship with laboratory-based objective cognitive tests (e.g., Refs. [3,7,17,27]; for a review see: [4]). In future studies, systematic comparison with different types of objective measurements would be crucial.

In summary, the present study aimed to validate the Hungarian version of CFQ, and it showed satisfactory results. CFQ items were described best with one single factor and the questionnaire was characterized with a high test-retest reliability and internal consistency. Furthermore, higher anxiety and depressive symptoms and lower well-being were associated with higher CFQ scores, and younger adults and women also tended to report more cognitive failures which fits to previous results. Our study indicates that similarly to the English version, the Hungarian translation of CFQ appears to be a well-useable tool to investigate everyday cognitive failures.

## Author contribution statement

Márta Volosin: Conceived and designed the experiments; Performed the experiments; Analyzed and interpreted the data; Contributed reagents, materials, analysis tools or data; Wrote the paper.

Emese Hallgató; Eszter Csábi: Conceived and designed the experiments; Performed the experiments; Analyzed and interpreted the data; Contributed reagents, materials, analysis tools or data.

## Funding statement

This study was supported by the University of Szeged Open Access Fund [5606].

## Data availability statement

Data will be made available on request.

## Declaration of interest’s statement

The authors declare no conflict of interest.
