# Supplementary material for: Validation of the Hungarian version of the Cognitive Failures Questionnaire (CFQ)
Source: Heliyon. 2023 Jan 10;9(1):e12910. doi: 10.1016/j.heliyon.2023.e12910 (PMC9853372; doi:10.1016/j.heliyon.2023.e12910)
Supplement: Multimedia component 1 [file mmc1.docx]

**Supplementary A**

**Item analysis of time stability and consistency of responses**

Because we aimed to investigate the temporal consistency not only of the summarized scale but also of each item, we run an analysis to describe the similarity of responses at the first and second completions. As there are no standardized item-wise comparison methods (note that test-retest reliability utilizes summarized scale sores and no single item scores), we considered several perspectives. We defined three potentially indicative values which could be sensitive to response inconsistencies over time: correlation coefficient, absolute difference and difference with sign between responses at the first and at the second completion.

First of all, as the first and second completions are expected to be correlated, and based on the level of measurement of items (ordinal) and the tied ranks, we computed *Kendall’s tau* for each item separately (presented in Table S1, column “Kendall’s tau”). A high positive correlation coefficient (τ > .5) indicates that when a participant responded with a high value at the first time on a certain item, they responded with a high value second time as well, and vice versa.

However, correlation per se does not fully indicate that the two responses were similar across the two completions. For example, it is possible that participants answered a question with a low value (e.g., 1 or 2) at the first time, then with a higher value (e.g., 3 or 4) at the second time or vice versa. In this case, the correlation coefficients can be high while the responses are obviously inconsistent between the two completions. To deal with this possible source of inconsistence, we computed the *absolute difference between the two responses* (responded at the first vs the second time, see Table S1, columns “Absolute difference”). In this case, it is irrelevant whether one responded with higher or lower value at the first or at the second time, but the point is the amount of difference between the two responses. This way one can identify items with the most different responses between the two time points.

Finally, despite that the absolute difference between the two responses can be regarded as an important indicator of instability of responses, it is not sensitive enough to identify systematic bias, that is whether respondents systematically give higher ratings at the first than at the second completion, or vice versa. This issue might be resolved by introducing a further indicator that takes into account the sign of the difference as well (higher ratings at first or at second time). This indicator was calculated by subtracting responses given at the second time from those given at the first time; negative values indicate higher ratings to the same item at the second time while positive values indicate higher ratings at the first time (presented in Table S1, columns “Difference”). In other words, it suggests a systematic shift towards higher or lower values in the first or the second time point. This shift might occur when the frequency of the observed behavior actually changes between the two time points or when the person becomes more aware of a behavior because of being explicitly asked, especially when they also know that they are going to be asked second time as well, leading to enhanced attention to the particular behavior and enhanced self-monitoring.

It is important to highlight that the above-mentioned indicators of item stability (correlation, absolute difference and difference between the two completions) are not independent of each other. For example, when averaging positive and negative difference values across the sample, it can occur that the *mean of difference between two responses* is around zero, that is, no systematic bias is present, but the mean absolute difference between responses is high. In this case we can talk about noise. Such noise might occur when for example, respondents find it difficult to judge whether a phenomenon occurs “quite often” or “very often”). Therefore, it is possible that one reaches a different conclusion about the same behavior, even though the frequency of the behavior did not actually change between the two time points.

High mean absolute difference between the two completions (noise) along with low mean difference also leads to reduced correlation coefficient but it does not affect bias necessarily. On the other hand, bias (systematically higher or lower values at first or second completion) is associated with large absolute difference. Therefore, it was considered being indicative of *noise* if Kendall’s tau correlation coefficient was lower than .5, and the mean of the absolute difference was higher or equal to 0.5, along with a mean difference lower than 0.2 or higher than -0.2. On the other hand, it was considered being indicative of *bias* when mean difference was larger or equal to 0.2 or lower than equal to -0.2.

Again, we had to come up with these thresholds ourselves because we couldn’t find any guidelines we could follow for this particular use. The interpretation of correlation coefficients varies between disciplines (Akoglu, 2018), and .7 indicates a strong correlation in psychology using Pearson’s or Spearman’s correlation. However, Kendall tau coefficients are usually smaller than Spearman’s coefficients calculated on the same datasets (Fredricks & Nelsen, 2007) so in this case a smaller value would already indicate a strong relationship. Furthermore, in ideal case these items should show a perfect relationship, so even an otherwise strong correlation (if it is a lot less than perfect) could be of concern for us. This is why we chose .5 as our threshold – anything below that is clearly less than perfect, even if it is still quite strong.

In an ideal case, the mean absolute differences between the response pairs (first time and second time) would be zero. We aimed to flag items where this was very obviously not the case. We thought that if the absolute differences had a mean of 0.5, that would mean that on average, half of the participants rated the items differently (with a difference of 1 score on the Likert scale) between the two occasions – and one half of all participants is anything but negligible. Since some people rated the same items even more differently between the two occasions (i.e., having a difference of 2-4 scores on the Likert scale), setting this threshold did not guarantee that we would observe differences in the case of 50% of the participants, as bigger differences increase the mean more than smaller differences. However, those also indicate higher instability on the particular items, so we sticked to this rule. In our case that resulted in flagging those items that got different ratings on the two occasions by at least 40% of our participants.

Lastly, looking at the raw differences (rather than their absolute values), our threshold of 0.2 would indicate that – on average – every fifth participant would systematically score higher by a score of 1 on the second occasion than on the first occasion – and vice versa for the negative values. We figured that this would indicate a non-trivial shift even if we take a possibly large noise into consideration, e.g., for every two participants scoring lower than previously by a score of 1, there would be three participants scoring higher by 1. In this example, the two (out of five) participants scoring lower and another two participants scoring higher would be simply noise that cancels each other out; the fifth participant however introduced a shift in the positive direction, and mathematically speaking, this would manifest as a shift of 0.2. Item statistics are presented in Table S1.

*Table S1. Item statistics including Kendall’s tau, mean and standard deviation of the absolute difference between the first and second completions irrespectively of the direction of the difference, and mean and standard deviation of the difference between the first and second completions.*

|  |  |  | Absolute difference  (first-minus-second) | | Difference  (first-minus-second) | |
| --- | --- | --- | --- | --- | --- | --- |
| Item number | Time-unstable indicator | Kendall’s tau | Mean | SD | Mean | SD |
| 1 |  | .59 | **0.60** | 0.70 | 0.15 | 0.91 |
| 2 |  | .66 | 0.47 | 0.65 | -0.03 | 0.80 |
| 3 |  | .54 | 0.47 | 0.69 | .009 | 0.83 |
| 4 |  | .65 | 0.35 | 0.71 | -0.03 | 0.79 |
| 5 |  | .65 | 0.20 | 0.43 | -0.01 | 0.48 |
| 6 |  | .56 | **0.54** | 0.76 | -0.03 | 0.93 |
| 7 |  | .56 | **0.70** | 0.80 | 0.15 | 1.06 |
| 8 |  | .63 | **0.53** | 0.64 | 0.02 | 0.83 |
| 9 |  | .54 | **0.66** | 0.74 | -0.08 | 0.99 |
| 10 |  | .61 | **0.61** | 0.69 | 0.15 | 0.90 |
| 11 |  | .58 | **0.66** | 0.75 | 0.07 | 0.99 |
| **12** | **Noise** | **.47** | **0.58** | 0.85 | -0.04 | 1.03 |
| 13 |  | .52 | **0.65** | 0.74 | 0.08 | 0.98 |
| 14 |  | .56 | **0.61** | 0.75 | -0.01 | 0.96 |
| 15 |  | .59 | **0.68** | 0.86 | 0.06 | 1.09 |
| 16 |  | .53 | 0.43 | 0.70 | -0.06 | 0.82 |
| 17 |  | .55 | **0.59** | 0.67 | 0.03 | 0.89 |
| 18 |  | **.47** | 0.42 | 0.63 | -0.04 | 0.76 |
| 19 |  | .61 | **0.59** | 0.65 | 0.04 | 0.88 |
| 20 |  | .53 | **0.65** | 0.77 | 0.13 | 1.00 |
| 21 |  | .60 | **0.67** | 0.69 | 0.01 | 0.96 |
| **22** | **Bias** | .55 | **0.56** | 0.70 | **0.20** | 0.88 |
| **23** | **Noise** | **.46** | **0.61** | 0.71 | 0.00 | 0.94 |
| **24** | **Noise** | **.44** | **0.64** | 0.82 | 0.08 | 1.03 |
| 25 |  | .56 | **0.56** | 0.78 | -0.01 | 0.96 |

Note: Time-unstable indicator is labeled as noise when low Kendall’s tau is accompanied with high absolute difference, and it is labeled as bias when both absolute difference and difference are considered as large. Items associated with noise or bias were considered as unstable (all values which suggested instability are highlighted in bold).

**References**

Akoglu, H. (2018). User’s guide to correlation coefficients. *Turkish Journal of Emergency Medicine, 18*(3), 91–93. <https://doi.org/10.1016/j.tjem.2018.08.001>

Fredricks, G. A., & Nelsen, R. B. (2007). On the relationship between Spearman’s rho and Kendall’s tau for pairs of continuous random variables. *Journal of Statistical Planning and Inference, 137*(7), 2143–2150. <https://doi.org/10.1016/j.jspi.2006.06.045>
