# Supplementary material for: Validation of the Hungarian version of the Cognitive Failures Questionnaire (CFQ)
Source: Heliyon. 2023 Jan 10;9(1):e12910. doi: 10.1016/j.heliyon.2023.e12910 (PMC9853372; doi:10.1016/j.heliyon.2023.e12910)
Supplement: Multimedia component 2 [file mmc2.docx]

**Supplementary B**

**Cognitive Failures Questionnaire**

The following questions are about minor mistakes which everyone makes from time to time, but some of which happen more often than others.

We want to know how often these things have happened to you in the past 6 months.

Please use the following scale: 4 – very often, 3 – quite often, 2 – occasionally, 1 – very rarely, 0 – never

|  | Very often | Quite often | Occasionally | Very rarely | Never |
| --- | --- | --- | --- | --- | --- |
| 1. Do you read something and find you haven't been thinking about it and must read it again? | 4 | 3 | 2 | 1 | 0 |
| 1. Do you find you forget why you went from one part of the house to the other? | 4 | 3 | 2 | 1 | 0 |
| 1. Do you fail to notice signposts on the road? | 4 | 3 | 2 | 1 | 0 |
| 1. Do you find you confuse right and left when giving directions? | 4 | 3 | 2 | 1 | 0 |
| 1. Do you bump into people? | 4 | 3 | 2 | 1 | 0 |
| 1. Do you find you forget whether you've turned off a light or a fire or locked the door? | 4 | 3 | 2 | 1 | 0 |
| 1. Do you fail to listen to people's names when you are meeting them? | 4 | 3 | 2 | 1 | 0 |
| 1. Do you say something and realize afterwards that it might be taken as insulting? | 4 | 3 | 2 | 1 | 0 |
| 1. Do you fail to hear people speaking to you when you are doing something else? | 4 | 3 | 2 | 1 | 0 |
| 1. Do you lose your temper and regret it? | 4 | 3 | 2 | 1 | 0 |
| 1. Do you leave important letters unanswered for days? | 4 | 3 | 2 | 1 | 0 |
| 1. Do you find you forget which way to turn on a road you know well but rarely use? | 4 | 3 | 2 | 1 | 0 |
| 1. Do you fail to see what you want in a supermarket (although it's there)? | 4 | 3 | 2 | 1 | 0 |
| 1. Do you find yourself suddenly wondering whether you've used a word correctly? | 4 | 3 | 2 | 1 | 0 |
| 1. Do you have trouble making up your mind? | 4 | 3 | 2 | 1 | 0 |
| 1. Do you find you forget appointments? | 4 | 3 | 2 | 1 | 0 |
| 1. Do you forget where you put something like a newspaper or a book? | 4 | 3 | 2 | 1 | 0 |
| 1. Do you find you accidentally throw away the thing you want and keep what you meant to throw away -- as in the example of throwing away the matchbox and putting the used match in your pocket? | 4 | 3 | 2 | 1 | 0 |
| 1. Do you daydream when you ought to be listening to something? | 4 | 3 | 2 | 1 | 0 |
| 1. Do you find you forget people's names? | 4 | 3 | 2 | 1 | 0 |
| 1. Do you start doing one thing at home and get distracted into doing something else (unintentionally)? | 4 | 3 | 2 | 1 | 0 |
| 1. Do you find you can't quite remember something although it's "on the tip of your tongue"? | 4 | 3 | 2 | 1 | 0 |
| 1. Do you find you forget what you came to the shops to buy? | 4 | 3 | 2 | 1 | 0 |
| 1. Do you drop things? | 4 | 3 | 2 | 1 | 0 |
| 1. Do you find you can't think of anything to say? | 4 | 3 | 2 | 1 | 0 |

**Kognitív Hibázás Kérdőív (CFQ – Cognitive Failures Questionnaire)**

A következőkben olyan apró hibákról kérdezzük, melyeket időről időre mindenki elkövet, azonban van, amit gyakrabban, van, amit ritkábban. Arra vagyunk kíváncsiak, hogy Önnel milyen gyakran fordultak elő ezek a hibák az elmúlt 6 hónap során. Kérjük, karikázza be a megfelelő számot!

4 – nagyon gyakran, 3 – viszonylag gyakran, 2 – néha, 1 – nagyon ritkán, 0 – soha

|  | Nagyon gyakran | Viszonylag gyakran | Néha | Nagyon ritkán | Soha |
| --- | --- | --- | --- | --- | --- |
| 1. Olvas valamit, és azon kapja magát, hogy nem azon gondolkodott, és újra el kell olvasnia? | 4 | 3 | 2 | 1 | 0 |
| 1. Azon kapja magát, hogy elfelejtette, miért ment a ház egyik feléből a másikba? | 4 | 3 | 2 | 1 | 0 |
| 1. Nem veszi észre a jelzőtáblákat az úton? | 4 | 3 | 2 | 1 | 0 |
| 1. Azon kapja magát, hogy összekeverte a jobb és a bal irányt, amikor útbaigazítást adott? | 4 | 3 | 2 | 1 | 0 |
| 1. Nekimegy embereknek? | 4 | 3 | 2 | 1 | 0 |
| 1. Azon kapja magát, hogy elfelejtette, lekapcsolta-e a lámpát vagy kikapcsolta-e a tűzhelyet, vagy hogy bezárta-e az ajtót? | 4 | 3 | 2 | 1 | 0 |
| 1. Nem figyel az emberek nevére, amikor találkoznak? | 4 | 3 | 2 | 1 | 0 |
| 1. Mond valamit, majd később rájön, hogy az sértő is lehetett? | 4 | 3 | 2 | 1 | 0 |
| 1. Nem hallja, hogy mások beszélnek Önhöz, mikor mással van elfoglalva? | 4 | 3 | 2 | 1 | 0 |
| 1. Elveszíti a türelmét, és megbánja? | 4 | 3 | 2 | 1 | 0 |
| 1. Napokig válasz nélkül hagy fontos leveleket? | 4 | 3 | 2 | 1 | 0 |
| 1. Elfelejti, merre kell fordulni egy Ön által ismert, de ritkán használt úton? | 4 | 3 | 2 | 1 | 0 |
| 1. Nem látja a boltban, amit akar, pedig az ott van? | 4 | 3 | 2 | 1 | 0 |
| 1. Azon kapja magát, hogy hirtelen azon töpreng, helyesen használt-e egy szót? | 4 | 3 | 2 | 1 | 0 |
| 1. Nehézséget okoz elhatároznia magát? | 4 | 3 | 2 | 1 | 0 |
| 1. Azon kapja magát, hogy elfelejti a megbeszélt időpontokat? | 4 | 3 | 2 | 1 | 0 |
| 1. Elfelejti, hova tett valamit, például egy újságot vagy egy könyvet? | 4 | 3 | 2 | 1 | 0 |
| 1. Azon kapja magát, hogy véletlenül kidobja, amit meg akart tartani, és megtartja, amit ki akart dobni - mint a példában: kidobja a gyufásdobozt, a használt gyufát pedig a zsebébe teszi? | 4 | 3 | 2 | 1 | 0 |
| 1. Elkalandozik, amikor figyelnie kellene valamire? | 4 | 3 | 2 | 1 | 0 |
| 1. Azon kapja magát, hogy elfelejti az emberek nevét? | 4 | 3 | 2 | 1 | 0 |
| 1. Elkezd csinálni egy dolgot otthon, és elterelődik valami más tevékenység felé (szándékán kívül)? | 4 | 3 | 2 | 1 | 0 |
| 1. Azon kapja magát, hogy nem emlékszik jól valamire, habár a “nyelve hegyén van”? | 4 | 3 | 2 | 1 | 0 |
| 1. Azon kapja magát, hogy elfelejti, mit ment venni a boltba? | 4 | 3 | 2 | 1 | 0 |
| 1. Elejt dolgokat? | 4 | 3 | 2 | 1 | 0 |
| 1. Azon kapja magát, hogy nem jut eszébe semmi, amit mondhatna? | 4 | 3 | 2 | 1 | 0 |
